# Canine companionship as a resilience factor: a quantitative inquiry into the impact of pet ownership on burnout mitigation among radiologists and radiographers

**DOI:** 10.7717/peerj.18110

**Published:** 2024-10-02

**Authors:** Dávid Sipos, Timea Jenei, Attila Pandur, Luca Anna Ferkai, Krisztina Deutsch, Arpad Kovács, Melinda Csima

**Affiliations:** 1 Department of Medical Imaging, Faculty of Health Sciences, University of Pécs, Kaposvár, Hungary; 2 “Moritz Kaposi” Teaching Hospital, Dr. József Baka Diagnostic, Radiation Oncology, Research and Teaching Center, Kaposvár, Hungary; 3 Department of Oxyology, Emergency Care, Faculty of Health Sciences University of Pécs, Pécs, Hungary; 4 Doctoral School of Health Sciences, University of Pécs, Pécs, Hungary; 5 Institute of Emergency Care and Health Pedagogy, Faculty of Health Sciences, University of Pécs, Pécs, Hungary; 6 Department of Oncoradiology, Faculty of Medicine, University of Debrecen, Debrecen, Hungary; 7 Institute of Education, MATE—Hungarian University of Agriculture and Life Sciences, Kaposvár, Hungary

**Keywords:** Radiology, Radiography, Radiologist, Radiographer, Burnout, COVID-19, Pet ownership, Mental health

## Abstract

**Background:**

The demanding nature of diagnostic imaging, coupled with the increasing workload and exposure to high-stress scenarios, underscores the pressing concern of burnout among radiologists and radiographers in modern healthcare settings. The objective was to investigate the interplay between family characteristics, workplace characteristics, pet ownership, and the occurrence of burnout.

**Methods:**

An online, quantitative, cross-sectional study with a non-random, purposive sampling method was carried out among Hungarian radiologists and radiographers from 1st of September to 1st of November 2022.

**Results:**

We examined the results of 406 responses predominantly from females (79.8%, *n* = 324), including 70.7% radiographers (*n* = 287). Cronbach’s alpha values for depersonalization (DP), emotional exhaustion (EE), and personal accomplishment (PA) were 0.74, 0.88, and 0.85, respectively. Average burnout scores were 8.35 (SD = 6.62) for DP, 26.26 (SD = 12.74) for EE, and 32.86 (SD = 9.52) for PA. DP demonstrated a balanced distribution (low: 35.7%, moderate: 27.3%, high: 36.9%). Conversely, EE and PA skewed towards high levels, with 52.5% (*n* = 213) and 49.5% (*n* = 201). Significant associations were found between gender and DP (*p* = 0.006), age (31–40 years) and DP/PA (*p* < 0.001; *p* = 0.004), absence of children and all burnout dimensions (*p* < 0.05), and pet ownership (*p* = 0.004) with lower EE, particularly for dog owners (*p* = 0.009). Occupation lacked a significant effect on burnout dimensions (*p* > 0.05). Employees without a second job had higher EE (*p* = 0.002) and lower PA (*p* = 0.008). Increasing healthcare experience correlated with decreased DP values (*p* = 0.001), while working over 40 h weekly negatively impacted all burnout dimensions (*p* ≤ 0.05). 15.5% (*n* = 63) exhibited signs of high burnout, with the age group 31–40 demonstrating the highest proportion (25.4%, *n* = 27) and significant associations with marital status, absence of children, pet ownership, private healthcare, 10–19 years in healthcare, and working over 40 h weekly.

**Conclusions:**

There is a pressing need for evidence-based strategies to alleviate burnout among radiologists and radiographers. There is a growing importance of recognizing the role of pets, especially dogs, as valuable companions for emotional support and stress relief. Implementing pet-friendly policies or therapy programs can contribute to a positive and supportive workplace, potentially mitigating burnout among essential frontline healthcare professionals.

## Introduction

Burnout among healthcare workers is of paramount concern as it not only compromises the well-being of dedicated professionals but also jeopardizes patient care. The demanding nature of the healthcare industry, coupled with long working hours and high-stakes responsibilities, intensifies the risk of burnout (Johnson et al., 2018; Bridgeman, Bridgeman & Barone, 2018). This chronic stress can lead to decreased job satisfaction, diminished quality of patient care, and a potential exodus of skilled professionals, ultimately undermining the overall effectiveness of the healthcare system (Rajamohan, Porock & Chang, 2019; Friganović et al., 2019).

Burnout among radiologists and radiographers is critically significant, as these professionals play a pivotal role in diagnostic and therapeutic decision-making (Chetlen et al., 2019; Fawzy et al., 2023). The intricate nature of medical imaging demands sustained focus and precision, making burnout a serious threat to the accuracy and timeliness of diagnoses. Moreover, the negative impact of burnout extends beyond individual well-being, potentially compromising patient outcomes and contributing to a shortage of skilled personnel in this vital healthcare domain (Chetlen et al., 2019; Fawzy et al., 2023; Sipos et al., 2023).

Research consistently shows high levels of burnout among radiologists, with factors such as working long hours and being female, being significantly associated with burnout (Bundy et al., 2020). This is further supported by a study in Australia and New Zealand, which found that radiographers and radiologists are all at risk of occupational burnout (Singh et al., 2017). The prevalence of burnout is also reported to be high among radiologists, with workplace and personal factors significantly contributing to stress (Parikh et al., 2020).

One of the leading contributors to burnout among radiology department workers is often the heavy workload and high volume of imaging studies (Bruls & Kwee, 2020). The continuous demand for interpreting and reporting diagnostic images, coupled with tight schedules and long hours, places significant stress on radiologists and radiographers, making it a primary factor in their burnout. In addition to workplace characteristics, family background also exerts a significant influence on an individual’s mental health (Bruls & Kwee, 2020; Harolds et al., 2016).

The COVID-19 pandemic has markedly increased the medical imaging workload due to a surge in diagnostic imaging demands, especially for chest imaging in the context of respiratory complications (Sotoudeh & Gity, 2021). Simultaneously, safety measures and operational challenges have slowed efficiency, creating backlogs and intensifying the workload for radiologists and radiographers (Sotoudeh & Gity, 2021; Wen et al., 2023).

Research on the incidence of burnout among radiographers and radiologists has identified several family characteristics that may contribute to this phenomenon. Radiographers with more than one child in their household reported a significantly better value of personal accomplishment, while those with a subjective assessment of poor financial status experienced higher levels of burnout. Identified family situation as a predictor of burnout among radiologists, with younger age and clinical focus being associated with higher burnout rates (Chetlen et al., 2019; Bundy et al., 2020). Bundy et al. (2020) found that identifying as female and working longer hours were significantly associated with burnout among interventional radiologists and cardiothoracic radiologists, respectively. These studies suggest that family characteristics such as number of children, financial status, and gender may play a role in the incidence of burnout among radiographers and radiologists (Bundy et al., 2020; Wen et al., 2023; Maresca et al., 2022).

Coping mechanisms against burnout include establishing work-life boundaries, practicing self-care routines, seeking social support, and incorporating stress-relief activities into daily life (Maresca et al., 2022). Pet ownership has shown a positive impact on mental health during the pandemic, providing companionship and reducing feelings of loneliness and isolation. The presence of pets has been linked to lower stress levels and improved emotional well-being, offering a source of comfort and stability during challenging times (Jones-Schenk, 2020; Jiang et al., 2023).

Regarding existing literature there is a need for comprehensive research to explore how specific family and workplace characteristics influence burnout among radiologists and radiographers. Additionally, further studies are required to evaluate the effectiveness and adoption of diverse coping mechanisms, such as pet ownership, in mitigating burnout within this professional group, especially in the context of increased pandemic-related stressors.

The aim of our survey was to examine the impact of family and workplace characteristics of radiologists and radiographers on individual burnout. During the survey, we placed particular emphasis on the presence of pets among the respondents.

## Materials and Methods

### Survey style and dissemination

Our survey was a quantitative, cross-sectional study with a non-random, purposive sampling method. The research was conducted in Hungary, and our online questionnaire was distributed to the electronic email addresses of radiographers and radiologists registered in the databases of the Hungarian Society of Radiographers, the Hungarian Chamber of Healthcare Professionals, and the Hungarian Society of Radiologists. Data collection took place between 1st of September and 1st of November 2022. Our online designed questionnaire was sent to the target groups in two installments over a period of 2 months. It was initially distributed in middle of September 2022, and then again in middle of October 2022.

### Target group

The target group consisted of radiologists and radiographers who had been actively working in patient care for at least one year during the COVID-19 pandemic. We excluded healthcare professionals on maternity or childcare leave and those who incompletely filled out the questionnaire.

### Survey instruments

Respondents in the study were informed about the purpose of the survey in the instructions at the beginning of the online questionnaire. The completion was voluntary, anonymous, and proceeded with the acceptance of the consent statement. Respondents had the option to interrupt their participation at any stage of the survey. By completing the questionnaire, respondents simultaneously consented to the research-oriented use of their responses.

### Measuring of burnout

The Maslach Burnout Inventory (MBI) has shown high internal consistency, with Cronbach’s alpha coefficients typically ranging from 0.70 to 0.90 across its subscales. This indicates that the items within each subscale are measuring the same underlying construct consistently. Studies have demonstrated that the MBI has satisfactory test-retest reliability over time, ensuring that it provides stable and consistent results when administered to the same individuals at different points in time (Ádám & Mészáros, 2012; Maslach, Jackson & Leiter, 1996).

The Maslach Burnout Inventory, consisting of 22 items, allowed respondents to indicate on a 7-point Likert scale for each item how characteristic the given response was for themselves. The scale included the following variables: never, a few times a year, once a month, several times a month, once a week, several times a week, every day. The questionnaire measures the level of burnout along three dimensions: emotional exhaustion (EE), depersonalization (DP), and personal accomplishment (PA). For items related to DP and EE, a high score indicates a high level of burnout, while for the dimension of PA, a low score indicates the extent of burnout (Ádám & Mészáros, 2012).

The categorization into three levels (low, moderate, or high) is determined based on the specified reference ranges in the MBI-HSS: for EE, the tiers are low (0–16), moderate (17–26), and high (≥27); for DP, the divisions are low (0–6), moderate (7–12), and high (≥13); and lastly, for PA, the breakdown is low (≤31), moderate (32–38), and high (≥39). Burnout is defined according to the updated Maslach-recommended criteria, which include “high EE and high DP” or “high EE and low PA”. The authors have permission to use this instrument from the copyright holders (Maslach, Jackson & Leiter, 1996) (Table 1).

**Table 1:** The values of the Maslach Burnout Inventory questionnaire by separate dimensions.

Dimension	Category	Value
Emotional exhaustion	Low	0–16
	Moderate	17–26
	High	≥27
Depersonalization	Low	0–6
	Moderate	7–12
	High	≥13
Personal accomplishment	Low	≤31
	Moderate	32–38
	High	≥39

### Self designed questionnaire

During our survey, we included a self-designed questionnaire containing closed-ended questions. This questionnaire covered socio-demographic, workplace information and specific questions related to the COVID-19 virus situation, formulated based on international literature. Creating questions regarding pet ownership involved a structured and methodical approach to ensure that the questions were clear, relevant, and capable of capturing the necessary information.

Based on the results of the pilot testing and feedback from experts and participants, we made necessary revisions to the questionnaire. This included rewording unclear items, adding or removing questions, and adjusting the response options to improve clarity and accuracy.

### Statistical analysis

The statistical processing of the incoming data was conducted using the Statistical Software Package for Social Sciences (SPSS) version 23.0. During our research, we employed descriptive statistics, two-sample t-tests, and analysis of variance (ANOVA). The combination of these statistical methods was chosen to ensure robust and appropriate analysis of the data collected during our research. Descriptive statistics provided a foundation for understanding the data, while two-sample t-tests and ANOVA allowed for hypothesis testing under normal distribution assumptions. When these assumptions were not met, the Mann-Whitney U test and Kruskal-Wallis test offered non-parametric alternatives to ensure the validity and reliability of our findings. The use of *p* ≤ 0.05 as a significance threshold was consistent with common research practices to determine statistically significant differences.

### Ethical consideration

Our research has been approved by the Medical Research Council—reference number: BMEÜ/253-1/2022/EKU.

## Results

### Descriptive results of the survey

A total of 406 responses were received. Women were overrepresented (79.8%; *n* = 324) in the survey. Radiographers comprised 70.7% of the respondents (*n* = 287). There was a nearly equal distribution across age groups. More than half of the respondents (66%; *n* = 268) were in a marital or partnership relationship, and there was a high number of singles (21.4%; *n* = 87). 52.2% of the respondents (*n* = 212) have children.

Considering the years spent working in healthcare, the majority had up to 9 years of work experience (*n* = 159, 39.2%). 90.6% of the respondents (*n* = 368) worked in state institutions. A total of 31.8% of those surveyed (*n* = 129) had a second job. The main job involved a predominantly 40-h workweek for the majority of respondents (77.1%; *n* = 313). More than half of the respondents (66.7%; *n* = 271) take on call duties, with most doing more than three shifts (*n* = 217; 53.4%).

Over half of the employees (53.4%; *n* = 217) have pets, with the majority (*n* = 90, 22.2%) having dogs. More than half of the radiographer (63.1%; *n* = 256) and radiologist respondents (60.3%; *n* = 245) believe that the COVID-19 pandemic has not contributed to increased societal appreciation for their professions.

### Results of the Maslach burnout inventory

Regarding the reliability of the questionnaire, the Cronbach’s alpha values were 0.74, 0.88, and 0.85 for the DP, EE, and PA dimensions, respectively.

Among the surveyed healthcare professionals, the average scores on the MBI questionnaire for burnout were 8.35 (SD = 6.62) for the DE subscale, 26.26 (SD = 12.74) for the EE subscale, and 32.86 (SD = 9.52) for the PA subscale.

In terms of the DP dimension categories, a nearly equal distribution was observed for the low (*n* = 145; 35.7%), moderate (*n* = 111; 27.3%), and high (*n* = 150; 36.9%) groups.

For the EE and PA dimensions, the majority fell into the high category, with *n* = 213 (52.5%) and *n* = 201 (49.5%) respondents, respectively (Table 2).

**Table 2:** The mean values of the Maslach Burnout Inventory and the severity of burnout.

		Maslach Burnout Inventory
Burnout dimension	Category	*n* (%)
**Depersonalization**	Low	145 (35.7)
	Moderate	111 (27.3)
	High	150 (36.9)
**OVERALL DEPERSONALIZATION MEAN**		**8.35 (SD = 6.62)**
**Emotional exhaustion**	Low	113 (27.8)
	Moderate	80 (19.7)
	High	213 (52.5)
**OVERALL EMOTIONAL EXHAUSTION MEAN**		**26.26 (SD = 12.74)**
**Personal accomplishment**	Low	104 (25.6)
	Moderate	101 (24.9)
	High	201 (49.5)
**OVERALL PERSONAL ACCOMPLISHMENT MEAN**		**32.86 (SD = 9.52)**

### The relationship between individual and family aspects and burnout

Men had significantly higher DP values than women (*p* = 0.006). Examining age, healthcare professionals aged 31–40 had significantly higher DP and significantly lower PA values (*p* < 0.001; *p* = 0.004). The absence of children had a negative impact on all three dimensions of burnout (*p* < 0.05). The presence of pets (*p* = 0.004), particularly among those with dogs (*p* = 0.009), was associated with significantly lower EE values (Table 3).

**Table 3:** The relationship between personal and family characteristics and burnout.

	*n* (%)	DP (mean ± SD)	EE (mean ± SD)	PA (mean ± SD)	High burnout (n, %)
*Gender*					
Male	82 (20.1)	10.14 ± 6.93	27.86 ± 13.33	33.57 ± 7.65	15; 18.2
Female	324 (79.9)	7.90 ± 6.47	25.85 ± 12.58	32.68 ± 9.94	48; 14.8
		*p* = 0.006			
		t = 2.75			
*Age*					
19–30 years	93 (22.9)	9.39 ± 7.25	26.40 ± 12.34	31.86 ± 8.04	12; 12.9
31–40 years	106 (26.1)	10.10 ± 6.09	28.39 ± 12.23	31.02 ± 11.25	27; 25.4
41–50 years	97 (23.4)	7.54 ± 6.36	25.70 ± 12.91	35.84 ± 8.80	15; 15.46
51+ years	110 (27.1)	6.51 ± 6.25	24.57 ± 13.29	32.76 ± 9.36	9; 8.1
		*p* < 0.001		*p* = 0.004	*p* = 0.010
		F = 6.82		F = 4.85	χ2 = 11.367
*Family* *status*					
Other	14 (3.4)	8.71 ± 6.62	29.42 ± 5.48	29.00 ± 11.20	2; 14.2
Single	87 (21.4)	10.11 ± 7.76	26.95 ± 12.66	31.45 ± 11.77	20; 22.9
Widowed	37 (9.1)	8.43 ± 6.40	28.05 ± 11.36	32.62 ± 8.97	3; 8.1
Married/living with spouse	268 (66.0)	7.76 ± 6.15	25.62 ± 13.21	33.55 ± 8.60	38; 14.1
*Children*					
Yes	212 (52.2)	7.22 ± 6.15	24.66 ± 13.01	34.33 ± 8.95	26; 12.2
No	164 (40.4)	10.38 ± 6.76	28.83 ± 11.85	30.09 ± 9.91	37; 22.5
		*p* < 0.001	*p* = 0.003	*p* < 0.001	*p* = 0.001
		F = 18.01	F = 5.84	F = 18.72	χ2 = 13.123
*Do you have any pets?*					
Yes	217 (53.4)	8.36 ± 7.16	24.53 ± 13.63	32.60 ± 10.89	14; 8.7
No	189 (46.6)	8.34 ± 5.95	28.22 ± 11.37	33.16 ± 7.68	49; 25.9
			*p* = 0.004		*p* = 0.007
			t = −2.919		χ2 = 7.168
*If yes, what type?*					
Dog	90 (41.4)	7.77 ± 7.21	23.69 ± 15.57	32.50 ± 11.71	2; 2.2
Cat	55 (25.3)	8.69 ± 8.60	24.36 ± 13.09	32.20 ± 12.03	6; 10.9
Dog and cat	44 (20.3)	9.02 ± 5.41	26.47 ± 13.39	34.63 ± 7.38	2; 4.5
Other	28 (12.9)	7.03 ± 6.02	23.82 ± 11.94	30.57 ± 10.43	3; 10.7
			*p* = 0.009		
			F = 2.46		

### The relationship between workplace characteristics and burnout

Occupation did not have a significant effect on the dimensions of burnout (*p* > 0.05). Employees who do not take on a second job had significantly higher EE values (*p* = 0.002) and significantly lower PA values (*p* = 0.008). With advancing years spent in healthcare, there is a significant decrease in DP values (*p* = 0.001). Working more than 40 h per week has a negative impact on all three dimensions of burnout (*p* ≤ 0.05) (Table 4).

**Table 4:** Work related characteristics and burnout.

	*n* (%)	DP (mean ± SD)	EE (mean ± SD)	PA (mean ± SD)	High burnout (*n*, %)
*Profession*					
Radiographer	287 (70.7)	8.44 ± 6.64	25.77 ± 12.77	32.96 ± 10.07	44; 15.3
Radiologist	119 (29.3)	8.15 ± 6.57	27.42 ± 12.66	32.63 ± 8.07	19; 15.9
*Full-time* *job*					
State	368 (90.6)	8.35 ± 6.69	26.14 ± 12.77	32.81 ± 9.45	53; 14.4
Private	38 (9.4)	8.42 ± 5.89	27.34 ± 12.63	33.34 ± 10.32	10; 26.3
					*p* = 0.001 χ2 = 11.145
*Second* *job*					
Yes	129 (31.8)	7.49 ± 6.26	23.40 ± 12.57	34.68 ± 9.87	19; 14.7
No	277 (68.2)	8.76 ± 6.75	27.59 ± 12.63	32.01 ± 9.25	44; 15.8
			*p* = 0.002	*p* = 0.008	
			t = −3.11	t = 2.65	
*Years* *spent* *in healhcare*					
1–9 years	159 (39.1)	9.47 ± 6.85	27.57 ± 11.87	31.35 ± 10.36	25; 15.7
10–19 years	72 (17.7)	9.23 ± 5.87	26.84 ± 13.38	32.18 ± 8.09	20; 27.7
20–29 years	60 (14.8)	8.00 ± 7.22	24.83 ± 13.91	36.31 ± 8.97	6; 10.0
30+ years	115 (28.3)	6.45 ± 6.00	24.81 ± 12.81	33.58 ± 8.99	12; 10.4
		*p* = 0.001		*p* = 0.005	
		F = 5.34		F = 4.40	
*Hours worked per week*					
Less than 40 h	67 (16.5)	8.40 ± 5.01	26.22 ± 12.19	31.98 ± 8.20	7; 10.4
40 h	313 (77.1)	7.98 ± 6.68	25.60 ± 12.72	33.47 ± 9.44	47; 15.0
More than 40 h	26 (6.4)	12.80 ± 7.98	34.30 ± 12.14	27.84 ± 12.11	9; 34.6
		*p* = 0.005	*p* = 0.004	*p* = 0.043	*p* = 0.001
		F = 6.55	F = 5.72	F = 4.61	χ2 = 13.377
*On-call shifts*					
No	135 (33.3)	7.91 ± 6.92	23.45 ± 15.56	31.85 ± 10.19	17; 12.5
Yes	271 (66.7)	8.58 ± 6.46	26.58 ± 12.23	33.37 ± 9.14	46; 16.9

### The presence of high burnout

The findings reveal that 15.5% (*n* = 63) of the respondents exhibited signs of high burnout.

Upon stratifying the data by age groups, it was discerned that the age bracket of 31–40 years exhibited the highest proportion of burned-out individuals, comprising 25.4% of this subgroup (*n* = 27), with a statistically significant association (*p* = 0.010). Marital status emerged as another factor influencing burnout, with 22.9% of singles (*n* = 20) demonstrating signs of burnout.

Furthermore, individuals without children displayed a burnout rate of 22.5% (*n* = 37), and those without pets exhibited a higher burnout prevalence at 25.9% (*n* = 49), both showing statistically significant associations (*p* = 0.001 and *p* = 0.007, respectively).

Occupational characteristics also played a role in burnout levels. In the private healthcare sector, the prevalence of burned-out individuals was 26.3% (*n* = 10) (*p* = 0.001). Among respondents with 10–19 years of experience in healthcare, the burnout rate was 27.7% (*n* = 20). The highest burnout rates were observed among those working more than 40 h per week, reaching 34.6% (*n* = 9), with a statistically significant association (*p* = 0.001).

## Discussion

In discussing the findings of this study on radiologists and radiographers’ burnout during the COVID-19 pandemic, it is crucial to delve into the implications of our research and the broader significance it holds for healthcare systems Previous studies on burnout among healthcare professionals in Hungary provide a baseline understanding. Examining how the pandemic affected burnout results could offer crucial insights into the specific impacts of COVID-19 on healthcare workers’ well-being and resilience in Hungary, contributing to a comprehensive assessment of the evolving challenges they face.

The high rates of EE observed among radiologist and radiographers can be indicative of chronic workplace stressors, highlighting the critical need for targeted interventions and organizational support structures to mitigate this prevalent issue. Additionally, a nuanced examination of PA rates within the same context underscores the complex interplay between individual resilience, job satisfaction, and the broader organizational climate, prompting further exploration into strategies that promote a balance between professional demands and personal fulfillment.

The pronounced DP rates among male radiologists and radiographers at the inception of their careers and at early ages necessitate a heightened awareness of potential contributing factors. In the absence of significant results, it can still be noted that women are emotionally less exhausted; however, their PA is lower. In our survey, the younger age group, specifically those aged 31–40, demonstrated the highest burnout scores in the dimensions of DP and EE. Similar to our findings, Adam et al. (2018) found significantly higher levels of DP among male doctors compared to females. However, they did not observe a significant relationship between male gender and low PA. In their survey, younger age was a predictor of EE.

International studies conducted among healthcare professionals also support the proposition that gender, being at the early stages of one’s career, young age, and lack of experience are all considered risk factors for burnout syndrome (Adam et al., 2018; Gayol & Lookingbill, 2022; Volpe et al., 2014; Singh et al., 2024).

In our study early-career male radiologists and radiographers experiencing heightened DP rates suggest the existence of formative challenges in the workplace, calling for research endeavors that delve into the organizational, interpersonal, and intrinsic factors influencing the development of DP at this critical stage of professional maturation.

The absence of children is correlated with increased EE and DP among healthcare professionals, reflecting the potential impact of family structure on the delicate balance between work and personal life. Individuals without children may encounter heightened challenges in coping with the emotional demands of their professions, contributing to elevated levels of burnout and reduced engagement with patients.

The moderating effect of workplace flexibility on the association between marital status (specifically, married healthcare workers without children) and burnout was examined. The sustained high level of burnout among married individuals was attributed to alterations in family roles, living conditions, and work circumstances. Facilitating strategies to uphold well-being within marital or familial contexts may prove efficacious in mitigating burnout, particularly amid the challenges posed by the COVID-19 pandemic (Chen et al., 2022; Maglalang et al., 2021).

The integral role of pets, viewed as familial entities, in the context of burnout and mental health is increasingly recognized, with studies suggesting that the companionship and emotional support provided by these animals can serve as protective factors against burnout and contribute positively to the mental well-being of their human caregivers (Ogata, Weng & Messam, 2023; Acquadro Maran et al., 2022).

The absence of pets and a lack of side jobs are associated with increased EE among individuals, suggesting that the absence of both companionship from pets and additional sources of personal fulfillment may contribute to heightened vulnerability to occupational stress and burnout. Understanding this connection emphasizes the importance of holistic well-being strategies that encompass both work-related and personal life factors to mitigate emotional exhaustion in various domains of individuals’ lives. Having a second job may reduce levels of EE by providing a sense of financial security and diversification of tasks, which can alleviate stress associated with primary job responsibilities. This diversification can introduce variety and new challenges, potentially making work more engaging and less monotonous. Additionally, the social interactions and different environments encountered in a second job can provide emotional support and a broader perspective, contributing to overall well-being.

Individuals aged 41–50 with 20–29 years of professional experience, particularly those who have children, exhibit notable connections with higher levels of PA. The accumulation of experience over two decades contributes to a refined skill set and expertise, while the presence of children may serve as a motivational factor, fostering a sense of responsibility that enhances one’s commitment to achieving professional goals. This age and experience cohort underscores the potential synergy between career longevity, family dynamics, and heightened levels of PA within this demographic.

Pereira et al. (2021), similarly to our findings, observed during the COVID-19 period that radiographers under the age of 30 exhibited low EE, while those aged 50 and above showed low DP values. The results of Giess et al. (2020) support that younger age, such as the category under 40 years old, is significantly associated with burnout.

In the survey conducted by Sipos et al. (2020), those with 1–5 and 16–20 years of experience showed higher EE and DP values. Similarly, in our own research, we observed a similar trend in the groups with 1–9 and 10–19 years of experience. In Pereira’s study among radiographers, 20–30 years of professional activity led to higher EE scores, while those with over 30 years of experience exhibited higher levels of PA. The results suggest that over the years, the trends of emotional exhaustion and DP decrease, which is explained by adapting to the work environment, accepting colleagues, and reducing emotional stress. This allows for a significant increase in PA values from the age of 40 onwards.

The deleterious impact of increased workload on burnout among healthcare workers was empirically substantiated. Elevated work demands were found to be significantly associated with heightened levels of burnout, highlighting the importance of mitigating excessive workloads to preserve the well-being of healthcare professionals (Ulfa, Azuma & Steiner, 2022; Portoghese et al., 2014; Rotenstein et al., 2023).

Regarding our study, working 40+ h per week has been consistently linked to an elevated risk of burnout among individuals, as prolonged and excessive working hours contribute to chronic fatigue, increased stress levels, and a diminished capacity for psychological recovery, ultimately undermining overall well-being. This negative effect underscores the importance of cultivating work-life balance and implementing strategies to mitigate the detrimental impact of extended work hours on mental health.

The observation of an approximately 15% high burnout rate among radiologists and radiographers signals a concerning prevalence of occupational stress and exhaustion within the healthcare imaging profession. Remarkably, only 8.7% of individuals who had pets at home exhibited high burnout rates, suggesting a potential protective effect of pet companionship on occupational well-being. This noteworthy correlation emphasizes the positive impact of having pets as a mitigating factor against burnout and underscores the importance of considering holistic approaches to promote mental health in professional settings.

Lass-Hennemann et al. (2020) investigated the relationship between dog ownership, psychopathological symptoms, and health-promoting factors among healthcare workers, police officers, and firefighters. Dog owners and non-dog owners exhibited similar levels of psychopathological anxiety and health-promoting factors.

The presence of a dog at home demonstrates a potential positive effect on burnout, with studies suggesting that the companionship and interactive nature of canine relationships may contribute to stress reduction, improved mental well-being, and enhanced resilience in individuals facing occupational challenges. The unique bond between humans and dogs holds promise as a supportive element in mitigating burnout and fostering a more resilient emotional state (Jensen et al., 2021; Machová et al., 2020). Individuals with dogs may experience lower emotional exhaustion and burnout values compared to cat owners due to several potential factors. Dogs typically require more interactive care, including regular exercise and outdoor activities, which can promote physical activity and reduce stress levels. The companionship provided by dogs is often more interactive and socially engaging, potentially enhancing emotional well-being and providing a source of comfort and support. The routine and responsibility of caring for a dog may create a structured daily schedule, which can contribute to a sense of purpose and reduce feelings of burnout associated with monotony or lack of variation in daily routines. These factors collectively suggest that the unique characteristics of dog ownership may contribute to lower levels of emotional exhaustion and burnout compared to owning a cat.

Implementing regular breaks and promoting a supportive workplace culture that acknowledges the importance of work-life balance can be effective coping mechanisms in reducing burnout among the radiology department. Encouraging team-building activities and creating spaces for open communication can enhance camaraderie and emotional support within the department, fostering a sense of collective well-being. Additionally, introducing stress management programs and providing resources for mental health support can further contribute to mitigating burnout and promoting a healthier work environment in the radiology department (Maresca et al., 2022; Menaldi et al., 2023; Rossi et al., 2023).

### Limitations

The cross-sectional design of our study among radiologists and radiographers may impede the establishment of causal relationships, as it captures data at a single point in time, making it difficult to discern the temporal sequence of events and ascertain the directionality of observed associations. The cross-sectional design also may limit the ability to assess the effectiveness of interventions or changes in workplace policies over time, hindering the exploration of factors that could potentially alleviate burnout among Hungarian radiologists and radiographers.

The observed gender differences in burnout are valid, though the widely different response sizes between males and females should be acknowledged.

The study may be susceptible to selection bias, as it includes respondents at a specific moment, possibly excluding those who have experienced significant burnout-related challenges and subsequently left the profession, leading to an incomplete representation of the population.

Also it is important to acknowledge that studies of this nature may have an inherent bias, as individuals experiencing higher levels of burnout are potentially more inclined to complete these surveys. This potential bias is not unique to this study but is a common consideration in all similar research.

## Conclusions

As we explore the nuances of burnout, our research not only sheds light on the challenges faced by radiologists and radiographers but also emphasizes the urgency for evidence-based strategies and interventions to alleviate burnout and sustain the well-being of these essential frontline healthcare professionals.

The presence of pets, particularly dogs, can serve as invaluable companions in reducing burnout by providing emotional support, stress relief. Incorporating pet-friendly policies, or pet therapy programs, can act as effective coping mechanisms, fostering a positive and supportive atmosphere that contributes to the overall well-being of employees and mitigates burnout.

Building on these insights, a targeted study could further investigate the impact of pet-friendly policies and pet therapy programs on burnout levels among healthcare professionals. This study could examine the specific benefits of pet interactions in reducing stress and emotional exhaustion; the feasibility and outcomes of implementing pet-friendly policies in healthcare settings; comparisons of burnout levels among healthcare professionals with and without access to pet therapy programs; longitudinal effects of sustained pet interaction on overall mental health and job satisfaction. Such a study would provide a deeper understanding of how incorporating pets into healthcare workers life can serve as a viable intervention for mitigating burnout and promoting well-being among radiologists, radiographers, and other frontline healthcare workers.

## Supplemental Information

10.7717/peerj.18110/supp-1 Supplemental Information 1 STROBE checklist.

10.7717/peerj.18110/supp-2 Supplemental Information 2 Self made questionnaire and MBI - in Hungarian and in English.

10.7717/peerj.18110/supp-3 Supplemental Information 3 Raw Data.

## Additional Information and Declarations

### Data Availability

The following information was supplied regarding data availability: The raw data is available in the Supplemental Files.
