# Supplementary material for: Canine companionship as a resilience factor: a quantitative inquiry into the impact of pet ownership on burnout mitigation among radiologists and radiographers
Source: PeerJ. 2024 Oct 2;12:e18110. doi: 10.7717/peerj.18110 (PMC11453153; doi:10.7717/peerj.18110)
Supplement: Supplemental Information 2 [file peerj-12-18110-s002.docx]

**Saját szerkesztésű kérdőív**

1. **Az Ön neme:**
2. Férfi
3. Nő
4. **Az Ön életkora: …… év**
5. **Az Ön magassága: …… cm**
6. **Az Ön testsúlya: …… kg**
7. **Az Ön foglalkozása**
8. radiológus
9. radiográfus (Bsc)
10. röntgen asszisztens és/vagy CT/MR operátor
11. **Dohányzik-e?**
12. igen, rendszeresen, napi … szál
13. igen, alkalmanként
14. nem
15. **A COVID-19 okozta járványhelyzet befolyásolta-e dohányzási szokásait?**
16. igen, többet dohányzom
17. igen, kevesebbet dohányzom
18. nem változott
19. **Fogyaszt-e kávét?**
20. igen, napi … csészével
21. igen, alkalmanként
22. nem
23. **A COVID-19 okozta járványhelyzet befolyásolta -e kávéfogyasztási szokásait?**
24. igen, több csésze kávét fogyasztok
25. igen, kevesebb csésze kávét fogyasztok
26. nem változott
27. **Melyik megyében dolgozik:**

………………………………………………………………………..

1. **Legmagasabb iskolai végzettsége, ami szakmájához köthető:**
2. alapfokú iskolai végzettség
3. középiskolai végzettség (szakközépiskola, gimnázium)
4. felsőfokú (felsőoktatási) szakképzésben szerzett bizonyítvány/ OKJ
5. főiskolai vagy felsőfokú alapképzésben (BA/BSc) szerzett oklevél
6. egyetemi vagy mesterképzésben (MA/MSc) szerzett oklevél
7. Phd/csc
8. **Jelenlegi munkahelyének jellege:**
9. közegészségügy
10. magánegészségügy
11. **Hány éve dolgozik az egészségügyben?**……………………………………………………………
12. **Hány órát dolgozik hetente főállásban, ügyelet nélkül?**
13. kevesebb, mint 40 óra
14. 40 és 50 óra között
15. több mint 50 órát
16. **Havonta hány ügyeletet vállal?**
17. egyet sem
18. 1-3
19. 3-5
20. 5+
21. **Jelenlegi családi állapota:**
22. házastársával/élettársával él
23. házastársával/élettársával és gyerekével él
24. házastársával/élettársával, gyerekével és szüleivel él
25. egyedülálló
26. egyéb családtagjával él, egyéb személlyel él
27. **Vannak-e gyerekei?**
28. igen, egy
29. igen, több is
30. nincs
31. **Volt-e valaha pozitív COVID tesztje?**
32. igen
33. nem
34. **Került-e karanténba COVID miatt?**
35. igen
36. nem
37. **Milyen speciális COVID-19 óvintézkedésekkel kapcsolatos képzésben részesült?**
    1. e-learning
    2. munkahelyi szimulációs továbbképzés
    3. tájékoztatókat és egyéb óvintézkedésekkel kapcsolatos oldalakat olvastam
    4. nem részesültünk külön kiemelt képzésben
38. **Munkavégzése során vizsgált-e gyanús/ igazolt COVID-19-es pácienst?**
    1. igen
    2. nem
39. **A COVID-19 ideje alatt került-e áthelyezésre más kórházba?**1. igen
    2. nem
40. **A COVID-19 ideje alatt került-e áthelyezésre kórházon belül?**
    1. igen
    2. nem
41. **A COVID-19 ideje alatt a közeli családjában, közeli barátai/kollégái körében volt-e igazolt koronavírusos?**
    1. igen, mind a családom, mind közeli barátaim, mind pedig kollégáim körében

2. igen, közeli családomban, vagy közeli barátaim körében

3. Igen, kollégáim körében

4. nem fordult elő a családom, mind közeli barátaim, mind pedig kollégáim körében COVID-19 fertőzés

1. **Van-e háziállata?**
2. igen, kutya
3. igen, macska
4. igen, egyéb: …………
5. nincs

**A Maslach Kiégés Leltár-Maslach Burnout Inventory kérdőív**

Olvassa el valamennyi állítást figyelmesen és döntse el, hogy érzett-e valaha így munkájával kapcsolatban! Kérjük, tegyen X-et a megfelelő rubrikába!

| **Milyen gyakran érzi a következőket?** | **soha** | **évente néhányszor** | **havonta maximum egyszer** | **havonta néhányszor** | **hetente egyszer** | **hetente többször** | **naponta** |
| --- | --- | --- | --- | --- | --- | --- | --- |
| l. Úgy érzem, hogy a munkámból érzelmileg kikapcsoltam  magam |  |  |  |  |  |  |  |
| 2. Úgy érzem, a munkanap végére elhasználódom. |  |  |  |  |  |  |  |
| 3.Fáradtnak érzem magam reggel, mikor felkelek, és szembe kell néznem egy újabb nappal a munkámban |  |  |  |  |  |  |  |
| 4. Könnyen megértem, hogy a rám bízott betegek mikor mit éreznek |  |  |  |  |  |  |  |
| 5. Úgy érzem, van néhány beteg, akit úgy kezelek, mintha nem is lenne külön egyéniségük (személytelen tárgyak lennének) |  |  |  |  |  |  |  |
| 6. Egész nap emberekkel foglalkozni igazán kimerítő és feszültségkeltő számomra. |  |  |  |  |  |  |  |
| 7.Nagyon eredményesen kezelem a velem kapcsolatban álló emberek (betegek, hozzátartozók) problémáját |  |  |  |  |  |  |  |
| 8.Kiégettnek érzem magam a munkámtól |  |  |  |  |  |  |  |
| 9. Úgy érzem, munkámon keresztül, pozitív hatással vagyok más emberek életére. |  |  |  |  |  |  |  |
| 10. Mióta ezt a munkát végzem, érzéketlenebb lettem az emberek iránt |  |  |  |  |  |  |  |
| 11. Aggódom, hogy ez a munka érzelmileg megkeményít. |  |  |  |  |  |  |  |
| 12. Nagyon energikusnak érzem magam. |  |  |  |  |  |  |  |
| 13.Frusztráltnak érzem magam a munkám során. |  |  |  |  |  |  |  |
| 14. Úgy érzem, túlságosan nagy energiát fektetek a munkámba |  |  |  |  |  |  |  |
| 15.Nem törődöm igazán azzal, hogy mi történik a betegekkel |  |  |  |  |  |  |  |
| 16. Túl sok stresszel jár számomra az, hogy közvetlenül emberekkel dolgozom |  |  |  |  |  |  |  |
| 17. A betegekkel könnyen ki tudunk alakítani egy oldott légkört |  |  |  |  |  |  |  |
| 18. A kórházi / rendelőintézeti munka után felüdülve érzem magam. |  |  |  |  |  |  |  |
| 19. Sok értékes dolgot végzek ebben a munkában. |  |  |  |  |  |  |  |
| 20. Úgy érzem, erőim végén tartok |  |  |  |  |  |  |  |
| 21. Munkám során az érzelmi problémákat nagyon nyugodtan kezelem. |  |  |  |  |  |  |  |
| 22.22. Úgy érzem, a velem kapcsolatban állók engem okolnak problémáik miatt. |  |  |  |  |  |  |  |

**Self designed questionnaire**

**1.** Your gender:

Male

Female

2. Your age: …… years

3. Your height: …… cm

4. Your weight: …… kg

5. Your occupation:

Radiologist

Radiographer (BSc)

6. Do you smoke?

Yes, regularly, …… cigarettes per day

Yes, occasionally

No

7. Has the COVID-19 pandemic affected your smoking habits?

Yes, I smoke more

Yes, I smoke less

No change

8. Do you consume coffee?

Yes, …… cups per day

Yes, occasionally

No

10. Has the COVID-19 pandemic affected your coffee consumption habits?

Yes, I drink more cups of coffee

Yes, I drink fewer cups of coffee

No change

10. In which county do you work: ……………………………………………………………………….

11. Highest educational qualification related to your profession:

Elementary school degree

Secondary school degree (vocational school, gymnasium)

Certificate/Degree from higher education (post-secondary vocational training or equivalent)

Bachelor's or equivalent degree (BA/BSc)

Master's or equivalent degree (MA/MSc)

Ph.D./DSc

12. Type of current workplace:

Public health

Private health

13. How many years have you been working in healthcare? ……………………………………………………………

14. How many hours do you work per week full-time, without on-call duty?

Less than 40 hours

Between 40 and 50 hours

More than 50 hours

15. How many on-call duties do you take on monthly?

None

1-3

3-5

5+

16. Current marital status:

Living with spouse/partner

Living with spouse/partner and child

Living with spouse/partner, child, and parents

Single

Living with other family member or other person

17. Do you have children?

Yes, one

Yes, more than one

No

18.. Have you ever had a positive COVID test?

Yes

No

19. Have you been in quarantine due to COVID?

Yes

No

20. What specific COVID-19 precautionary training have you received?

e-learning

Workplace simulation training

Read information and other precautions-related materials

Did not receive specific highlighted training

21. Have you examined a suspected/confirmed COVID-19 patient during your work?

Yes

No

22. Have you been transferred to another hospital during COVID-19?

Yes

No

23. Have you been transferred within the hospital during COVID-19?

Yes

No

24. During COVID-19, have there been confirmed cases of coronavirus in your close family, close friends, or colleagues?

Yes, in both my family and close friends and colleagues

Yes, in my close family or close friends

Yes, among my colleagues

No COVID-19 infection occurred in my family, close friends, or colleagues

25. Do you have a pet?

Yes, a dog

Yes, a cat

Yes, other: …………

No

Az űrlap teteje

MBI - Human Services Survey - MBI-HSS: Copyright ©1981 Christina Maslach & Susan E. Jackson.

All rights reserved in all media. Published by Mind Garden, Inc., www.mindgarden.com

MBI Human Services Survey

How often: 0 1 2 3 4 5 6

0 Never

1 A few times a yearor less

2 Once a month or less

3 A few times a month

4 Once a week

5 A few times a week

5 Every day

Statements:

1. _________ I feel emotionally drained from my work.

2. _________ I feel used up at the end of the workday.

3. _________ I feel fatigued when I get up in the morning and have to face another day on the job.

4. _________ I can easily understand how my recipients feel about things.

5. _________ I feel I treat some recipients as if they were impersonal objects.

6. _________ Working with people all day is really a strain for me.

7. _________ I deal very effectively with the problems of my recipients.

8. _________ I feel burned out from my work.

9. _________ I feel I'm positively influencing other people's lives through my work.

10. _________ I've become more callous toward people since I took this job.

11. _________ I worry that this job is hardening me emotionally.

12. _________ I feel very energetic.

13. _________ I feel frustrated by my job.

14. _________ I feel I'm working too hard on my job.

15. _________ I don't really care what happens to some recipients.

16. _________ Working with people directly puts too much stress on me.

17. _________ I can easily create a relaxed atmosphere with my recipients.

18. _________ I feel exhilarated after working closely with my recipients.

19. _________ I have accomplished many worthwhile things in this job.

20. _________ I feel like I'm at the end of my rope.

21. _________ In my work, I deal with emotional problems very calmly.

22. _________ I feel recipients blame me for some of their problems
